# Psychosocial Burden and Supportive Care Needs of Informal Caregivers in Specialist Palliative Care: Protocol of a Multicenter Longitudinal Cohort Study to Identify Trajectories and Validate the Multidimensional Screening Tool CAREPAL-8

**DOI:** 10.2196/78076

**Published:** 2026-07-31

**Authors:** Clara Haufschild, Karin Oechsle, Antonia Zapf, Anne Daubmann, Philipp Weber, Holger Schulz, Anneke Ullrich

**Affiliations:** 1 Palliative Medicine, Department of Oncology, Hematology and BMT, University Medical Center Hamburg-Eppendorf, Martinistraße 52, Hamburg, 20246, Germany, 49 40741055690; 2 Institute of Medical Biometry and Epidemiology, University Medical Center Hamburg-Eppendorf, Hamburg, Germany; 3 Department of Medical Psychology, University Medical Center Hamburg-Eppendorf, Hamburg, Germany

**Keywords:** specialist palliative care, informal caregivers, psychosocial burdens, supportive care needs, trajectory, screening

## Abstract

**Background:**

Informal caregivers (ICs) of patients with advanced incurable diseases experience substantial psychosocial burden and unmet needs. However, efficient screening tools to identify ICs at risk of developing clinically relevant psychosocial problems are lacking. Additionally, most existing studies on burden and needs use cross-sectional designs, failing to capture the dynamic and evolving nature of caregiving.

**Objective:**

This study aims (1) to explore the trajectories of ICs’ psychosocial burden and supportive care needs during specialist palliative care (SPC) and factors associated with these trajectories and (2) to validate the 8-Item Screening Tool for Family Caregiver Burden in Palliative Care (CAREPAL-8), an 8-item multidimensional screening tool developed to categorize ICs into clinically relevant risk groups regarding dimensions of burden.

**Methods:**

This prospective, multicenter, longitudinal cohort study is conducted across 16 study centers in Germany, involving palliative care wards, multiprofessional SPC teams in hospitals, and specialist palliative home care teams. Data are collected upon the patient’s admission to SPC (baseline) and weekly for up to 10 weeks, using validated questionnaires and the CAREPAL-8. Based on a priori sample size calculation, we aim to collect data from 510 ICs at baseline and on at least 1 additional follow-up measurement point. To minimize participant burden, a planned missing data design is used for follow-up questionnaires. Data analyses will include growth mixture models for study aim 1 and determination of convergent validity and sensitivity to change of the CAREPAL-8 for study aim 2.

**Results:**

Data collection commenced on July 31, 2023. Results are expected from March 2026.

**Conclusions:**

This study aims to provide comprehensive insights into the trajectories of IC burden and needs in SPC. The validation of the CAREPAL-8 could offer a brief screening tool to classify ICs according to different risk profiles, enabling targeted support. Furthermore, the planned missing data design may enhance data quality while reducing participant burden, potentially serving as a model for similar studies in vulnerable populations.

## Introduction

Advanced incurable diseases affect not only the patients but also those around them [1]. Family members and other related persons such as close friends often play an important role in providing unpaid care and support and are therefore considered informal caregivers (ICs) [2]. However, ICs experience their own burdens and needs [1]. Despite the recognized importance of caregiver burden in palliative care, a clear and consistent definition is still missing. As noted in the review by Choi and Seo [3], caregiver burden is a multidimensional concept that encompasses physical symptoms, psychological distress, strained social relationships, spiritual distress, and financial problems resulting from caregiving responsibilities. If this caregiver burden remains unresolved, this can lead to psychiatric disorders, impaired physical health status, and diminished quality of life [3]. Furthermore, international studies show high rates of unmet supportive care needs of ICs, for example, regarding information, support, or communication [4-6]. For these reasons, palliative care represents a “family-centered” approach, including both the patient and ICs as a “unit of care” [7].

To provide optimal care for ICs, it is essential to systematically identify their burden and supportive care needs. Several tools are available, either developed and validated specifically for ICs in the palliative context (eg, the Caregiver’s Burden Scale in End-of-Life Care [8]), instruments initially developed for other populations but validated for ICs (eg, Distress Thermometer [DT] [9]), or generic instruments developed for more broad populations (eg, Patient Health Questionnaire Depression Module [PHQ-9] [10,11]). Many of these tools are unidimensional, with items measuring the same dimension of burden in terms of, for example, psychological distress or family conflict. To gain a comprehensive understanding of different aspects of caregiver burden, it is therefore inevitable to administer a set of tools simultaneously [12]. However, given the vulnerable situation of ICs and the time constraints in clinical settings, the aim is to use an efficient multidimensional screening with a minimal number of items [13]. While there are also multidimensional instruments, for example, the Caregiver Reaction Assessment [14], there remains a gap in efficient instruments that help clinicians identify ICs at risk of developing clinically relevant psychosocial problems in daily practice [12,13]. To address this, our working group [12] has developed the 8-Item Screening Tool for Family Caregiver Burden in Palliative Care (CAREPAL-8), a multidimensional screening tool that categorizes ICs into 4 clinically relevant risk groups, offering tailored recommendations for support. However, the CAREPAL-8 has not been validated yet.

Despite the recognized prevalence of unmet needs and burdens experienced by ICs during palliative care [1,15-17], systematic longitudinal studies measuring trajectories of burdens and needs are still limited. Compared to cross-sectional studies, longitudinal research captures the dynamic process of caregiving over time [18]. Understanding joint trajectories of burden and needs, as well as factors associated with these trajectories, would facilitate the development and targeting of effective support programs [19]. Existing studies show various burden trajectories. Both quantitative and qualitative studies have identified two trajectories of depressive symptoms and caregiver burden: (1) persistent or stable and (2) increasing over time [20,21]. While some studies found a nonlinear increase in caregiver burden over time up to the patient’s death [19] or a substantial increase in burden and depression in the terminal stage of illness [22], other studies suggest that burden does not significantly increase as death approaches [23] or remains relatively constant over a 2-year study period [18]. Many factors related to burden and their trajectories have been discussed, including low economic status or insufficient financial resources, patient condition, and contextual factors [3,18-20,22-24]. With regard to ICs’ needs, a review of predominantly cross-sectional studies indicates a wide range of unmet care needs in ICs of patients with advanced cancer, emphasizing the importance of prospective longitudinal data [4]. Longitudinal qualitative studies, along with research analyzing cross-sectional retrospective data, demonstrate changes in needs of ICs over time, influenced by both medical and nonmedical developments [25-27]. Taken together, existing palliative care research suggests that ICs’ burdens and needs can change during the caregiving process, depending on various factors. When examining these studies, it is crucial to consider the cultural context, differences in palliative care settings, and the disease spectrum. Studies show that the evolution of caregiver burden differs between ICs caring for patients with cancer versus patients with nononcological diseases [28], as well as between inpatient and outpatient palliative care settings [29]. To our knowledge, no study has yet investigated the trajectories of burden and needs across different settings of specialist palliative care (SPC) systematically and longitudinally in short time intervals.

With this study, we aim, first, to record longitudinal data on the burden and supportive care needs of ICs in the course of SPC. The data will be analyzed with regard to joint trajectories of burden and supportive care needs. If specific trajectories can be identified, their characteristics, frequencies, as well as associated factors will be examined. Additionally, comparisons between different settings of SPC will be conducted. Second, we seek to validate the new multidimensional screening tool CAREPAL-8.

## Methods

### Overview

This study protocol was written under consideration of the SPIRIT (Standard Protocol Items: Recommendations for Interventional Trials) guideline [30]. As this study is categorized as a cohort study without an intervention, only relevant items were included. The SPIRIT checklist is displayed as Checklist 1. An adapted version of the SPIRIT schedule as well as the items from the World Health Organization Trial Registration Data Set are displayed in Multimedia Appendices 1 and 2.

### Study Design and Setting

This 3-year cohort study on the psychosocial burden and supportive care needs of ICs in SPC in Germany implements a prospective, multicenter, longitudinal design with up to 11 measurement points using self-report questionnaires. To minimize the burden of repeated data collection in this vulnerable population, a planned missing data design is implemented for all follow-up measurement points [31,32]. This methodological approach allows item allocation between participants, resulting in shorter questionnaires and reduced completion time per participant [32]. Further details on the implementation of the planned missing data design used in this study are provided in the Follow-Up Questionnaires (t1-t10) and Implementation of the Planned Missing Data Design section.

A total of 16 university hospitals and nonacademic facilities across Germany, hereinafter referred to as study centers, are participating in data collection. The consortium management lies with the University Medical Center Hamburg-Eppendorf, hereinafter referred to as the leading center. The study centers are located in 7 federal states of Germany, representing both urban and rural areas. Three common settings of SPC in Germany are involved: (1) palliative care wards, (2) multiprofessional SPC teams in hospitals, and (3) specialist palliative home care teams. According to the typology suggested by Wikert et al [33], all 3 settings employ multiprofessional staff, most of them with palliative care qualification. Palliative care wards are independent hospital wards, while multiprofessional SPC teams in hospitals operate hospital-wide. Specialist palliative home care teams can either be affiliated with a hospital or operate independently. Some of the study centers include more than 1 setting so that data collection is performed on 11 palliative care wards, in 5 multiprofessional SPC teams in hospitals, and in 8 specialist palliative home care teams. An overview of the 16 study centers, including their locations and the SPC settings they cover, is provided in Multimedia Appendix 3.

### Participants

During the study period, each patient admitted to a participating study center is assessed for the eligibility of one IC to participate. Inclusion criteria for ICs are being aged 18 years and older and being a close relative or a significant person in the patient’s life, for example, family member, friend, or other person with a close relationship to the patient, who provides care or support [2]. Exclusion criteria include the patient’s imminent death, legal guardianship without a personal connection to the patient, and insufficient German language skills of the IC, as the questionnaires are only available in German. There are no restrictions regarding the patient’s disease type, including oncological and nononcological progressive diseases. The disease type will not be assessed, as valid ascertainment would either require patient consent for medical record access or rely solely on IC-reported information. Prior treatment in SPC is allowed and will be included as a covariate in the analyses. Reasons for exclusion or nonparticipation of ICs are systematically documented by study staff throughout the recruitment period using a standardized form. This documentation allows for estimating how many ICs did not participate due to high psychosocial burden, which is one of the study’s outcomes. A priori sample size calculation was conducted, as detailed in the Sample Size Calculation section.

### Data Collection

After the patient’s admission to one of the participating study centers, ICs are consecutively recruited in person or by telephone by trained study staff (for details about the training, see Data Quality Assurance section). ICs are provided with information about the study, including its aims, procedures, and data privacy details, in accordance with German legal regulations. Written informed consent is obtained from ICs prior to data collection. Baseline data (t0) were initially planned to be collected within 3 days after the patient’s admission to SPC in order to capture the burden and needs of ICs at initiation of SPC. After testing the recruitment procedures in the clinical setting, the permissible baseline assessment period was extended to 7 days to reduce data loss, while still aiming to complete assessments within the original 3-day time frame whenever possible. Counting from the date of the ICs’ consent to participate in the study, the remaining 10 questionnaires are completed weekly (t1-t10). The date of consent was selected as the starting point for scheduling weekly follow-up measurements to ensure timely preparation and distribution of the questionnaires, regardless of when the baseline questionnaire is returned. The rationale for the weekly follow-up interval aligns with the regular evaluations required for the patient burden and needs assessment for health insurance billing for SPC in Germany. In accordance with the “unit of care” approach in palliative care, we aim to use the same interval for ICs.

**Figure 1.**
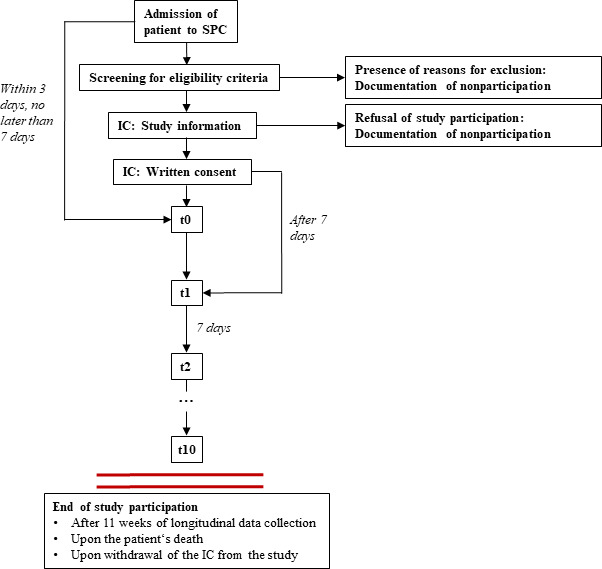
Recruitment and data collection procedure. IC: informal caregiver; SPC: specialist palliative care; t0: baseline; t1-t10: follow-up measurement points.

Questionnaires are available exclusively in paper-pencil format and are either handed to the ICs in person, left at the patient’s care location, or sent by mail. In exceptional circumstances (eg, if a willing IC is on vacation abroad), the questionnaire may be completed via a structured telephone interview conducted by study staff, with this process duly documented. If necessary, the ICs may receive assistance from study staff or another person in completing the questionnaire; any such support is recorded in the respective questionnaire. These factors may be accounted for in the analyses as potential covariates. All pseudonymized questionnaires are returned by the ICs via prepaid envelopes or by study staff via mail, fax, or scan to the leading center for data entry and evaluation. Secured cloud systems with pseudonymized shared charts to document the distribution and receipt of questionnaires are used to coordinate data collection between study centers. Participation in the study will end either after 11 weeks of longitudinal data collection, upon the patient’s death, or if the IC withdraws from the study. Figure 1 illustrates the recruitment and data collection procedure.

### Measures for Quality and Ethical Considerations

#### Data Quality Assurance

Various measures are implemented to ensure high data quality throughout the study. A key measure involves holding regular project meetings with the leading center and all staff involved in the study—including researchers and members of the palliative care teams—across all 16 study centers. Kick-off meetings are held both online and in person at the beginning of the study. Prior to data collection, all study team members receive standardized training on data collection and quality. This training, developed from years of experience with recruiting ICs in palliative care [34,35], lasts 90 minutes and is conducted by the leading center of this study. Trainings can be conducted online with staff from the same or different centers or on-site at the study centers. Written documentation of the training is provided. Additionally, individual or combined meetings are held at each study center to address specific conditions and requirements.

Additional mandatory and needs-based project meetings are scheduled throughout the data collection process to review insights, address uncertainties and challenges faced by the participating study centers, and share successful strategies. These different project meetings can help to increase involvement in the study and to minimize gatekeeping and insecurities in data collection [36].

To ensure secure and timely communication regarding the documentation of relevant information, multiple data transmission methods are available (eg, secured cloud systems and fax machines). All study processes and communication procedures have been verified and documented in a data protection plan and data protection impact assessment.

The study is overseen by a steering committee responsible for monitoring study progress and regularly evaluating data quality and procedural adherence. Meetings are convened 1 to 2 times per month, depending on the current phase of the study. The committee comprises CH, AD, PW, HS, and AU, with regular contributions from additional coauthors of this paper. It represents an interdisciplinary perspective, drawing on expertise in medicine, psychology, sociology, mathematics, and statistics. Any protocol modifications deemed necessary will be formally communicated to all relevant stakeholders, including study staff across participating centers, the study sponsor, and the responsible ethics committees. At the time of submission, the study protocol is version 1.3, dated March 31, 2025.

All physical study material (eg, signed informed consent forms) is securely stored in locked rooms at the study centers, while digitalized data are hosted on protected servers at the leading center. Data entry is centralized at the leading center, with regular duplicate entries performed on approximately 10% of baseline questionnaires and 2% of follow-up questionnaires, both randomly selected. Before data entry, study staff receive training on the standardized SPSS files and data entry protocols. To ensure consistency and accuracy, written documentation—including detailed codebooks and data entry guidelines for each questionnaire—is provided. Prior to data evaluation, the data will be cleaned in accordance with a set of predefined, written rules established a priori. This process includes range and plausibility checks as well as the review of handwritten entries. Access to the cleaned dataset will be restricted to researchers affiliated with the leading center and the designated scientific project leads at the participating study centers. Data will only be shared in pseudonymized or anonymized form to ensure participant confidentiality. Any additional exploratory analyses proposed by study centers will require prior review and approval by the leading center to ensure alignment with the overall study objectives and data governance protocols.

#### Ethical Considerations

Given that this study is conducted in the vulnerable setting of SPC, several ethical considerations were made. The study follows the Methods of Researching End of Life Care by the UK Medical Research Council and National Institutes of Health Research in its planning and implementation [37]. Primary ethics approval was granted by the ethics committee of the Medical Association of Hamburg, Germany (2022‐100878-BO-ff), and, if required, secondary ethics approvals were obtained from the local committees of the other study centers.

During the data collection period, a regular telephone consultation hour with a psychologist in the leading center is offered for ICs to mitigate any potential study-induced burden. The consultation times and contact details are provided on each questionnaire.

A significant proportion of patients is expected to die within a few weeks after admission to SPC in Germany [38-40]. As patient death represents the end of this study, the information of patient death is documented in order to stop the dispatch of questionnaires. Additionally, if the patient dies in a facility not participating in the study, ICs are asked to report the death via the follow-up questionnaires, which include a sensitively phrased option for documenting the death. ICs are informed that no further questionnaires need to be completed. In the event of a patient’s death during the study, condolence cards are sent by the study team. Thank-you cards are sent if an IC drops out due to other reasons or concludes the study period.

### Instruments

#### Overview

To record ICs’ burden and needs, the baseline questionnaire and follow-up questionnaires consist of the following validated scales (German versions): DT of the National Comprehensive Cancer Network [41,42], Generalized Anxiety Disorder 7-item scale (GAD-7) [43,44], PHQ-9 [10,11,45], Short-Form Health Survey (SF-8) [46,47], Family Inventory of Needs (FIN) [48,49], and Oslo Social Support Scale (OSLO-3) [50,51]. While the FIN was specifically developed for assessing the needs of ICs of severely ill people, most of the questionnaires are generic (GAD-7, PHQ-9, and SF-8). The DT was originally developed for patients with cancer, but is also validated for the screening of ICs [9]. Previous studies have shown the feasibility of similar questionnaire surveys with ICs in SPC [34,52]. To align with the weekly assessment schedule, the recall periods of the GAD-7 and PHQ-9 were adapted from 2 weeks to 1 week. This adjustment was made to ensure consistency with the measurement intervals and to avoid overlap between consecutive assessments.

The instrument to be validated, the CAREPAL-8, is also part of each questionnaire. The CAREPAL-8 is a newly developed multidimensional 8-item screening instrument of IC burden in palliative care [12]. It was developed in a data-driven approach and consists of single items of the GAD-7, PHQ-9, SF-8, and FIN [12]. For the analysis, each item is dichotomized. By summarizing 4 items each, 2 indices are calculated: index 1 measuring psychopathological symptoms and quality of life (PSYQOL) and index 2 measuring the fulfillment of needs (NEEDS) [12]. By combining both indices, ICs are allocated to one of four groups with different risk profiles: (1) currently stable ICs (index 1=0 and index 2≤1), (2) ICs with unmet needs (index 1=0 and index 2≥2), (3) psychologically burdened ICs (index 1≥1 and index 2≤1), and (4) high-risk ICs (index 1≥1 and index 2≥2) [12]. For each group, the authors included clinical recommendations for further IC assessment and support [12]. Table 1 gives an overview of the included scales.

**Table 1.** Included scales^a^.

Outcome	Questionnaire or scale	Description	Analyses
Distress	Distress Thermometer (DT) of the National Comprehensive Cancer Network	A 1-item analog scale that measures the subjective distress within the last week ranging from 0=no distress to 10=extreme distress [41,42]. Additionally, an adapted list of 24 problems that possibly cause the distress is included (answer “yes” or “no”) [52].	For the use of the German version, a cutoff value of ≥5 is recommended as an indicator for clinically relevant distress that needs professional clarification and support [42].
Anxiety	Generalized Anxiety Disorder 7-item scale (GAD-7)	Measures the frequency of core symptoms of generalized anxiety disorder within the past 2 weeks according to the Diagnostic and Statistical Manual of Mental Disorders, Fourth Edition (DSM-IV) [43,44]. Due to the weekly survey frequency, the assessment period in this study refers to the past week. In total, 7 items are rated on a 4-point Likert scale ranging from 0=not at all to 3=nearly every day.	Calculation and analysis of a sum score (ranging from 0 to 21). Cutoffs are 0‐4=absence, 5‐9=mild, 10‐14=moderate, and ≥15=severe anxiety levels. Standard values for the German general population are available [44].
Depression	Patient Health Questionnaire Depression Module (PHQ-9)	Measures the frequency of core symptoms of depression within the past 2 weeks according to DSM-IV [10,11,45]. Due to the weekly survey frequency, the assessment period in this study refers to the past week. In total, 9 items are rated on a 4-point Likert scale ranging from 0=not at all to 3=nearly every day.	Calculation and analysis of a sum score (ranging from 0 to 27). Cutoffs are 0‐4=absence, 5‐9=mild, 10‐14=moderate, and ≥15=severe depressive symptom levels. Standard values for the German general population are available [11].
Health-related quality of life	Short-Form Health Survey (SF-8)	Validated short form of the Health Survey Form-36 [46,47]. Consists of 8 items, each of which measures one aspect of health-related quality of life. Each item is answered on a different 5- or 6-point Likert scale. Due to the weekly survey frequency, the assessment period in this study refers to the past week.	Each scale value is linearly transformed. Two summary component scores can be calculated: physical and mental quality of life. Age- and gender-specific standard values for the German general population are available [47].
Needs	Family Inventory of Needs (FIN)	Measures the needs of family caregivers of severely ill patients in terms of their current importance and fulfillment [48,49]. Contains 20 items that are each rated on 2 subscales: FIN-Importance, ranging from 1=not important to 5=extremely important, and FIN-Fulfillment, ranging from 0=not met, 0.5=partly met to 1=met [49].	The scale FIN-Importance is dichotomized into 0=not or somewhat or moderate vs 1=very or extremely important. The scale FIN-Fulfillment is dichotomized into 0=not or partly met and 1=met. In addition to the sum scores of the number of important and unfulfilled needs (scale 0‐20 in each case), corresponding values can also be calculated for 4 dimensions of the FIN: basic information (scale 0‐4), information on treatment and care (scale 0‐7), support (scale 0‐7), and patient comfort (scale 0‐2) [53]. The German version was used with the courtesy of Schur et al [49].
Social support	Oslo Social Support Scale (OSLO-3)	OSLO-3 is a 3-item instrument that measures the current extent of perceived social support on a 4- or 5-point Likert scale with respect to the number of close confidants, the concern from others, and the accessibility of practical help from neighbors [50].	Calculation and analysis of a sum score (ranging from 3 to 14). Suggested cutoffs are 3‐8=poor social support, 9‐11=moderate social support, and 12‐14=strong social support [54].
Multidimensional informal caregiver (IC) burden	8-Item Screening Tool for Family Caregiver Burden in Palliative Care (CAREPAL-8)	Newly developed multidimensional 8-item screening instrument of IC burden to be validated in this study [12]. Consists of single items from established scales that had been identified to be most predictive for allocation of ICs to 4 groups with distinct risk profiles. Includes items from GAD-7 (item 2), PHQ-9 (item 4), SF-8 (item 4 and item 8), and FIN (item 6, item 11, item 16, and item 17).	Two indices are calculated. Index 1 for psychopathological symptoms and quality of life (PSYQOL), consisting of the selected items of the GAD-7, PHQ-9, and SF-8. Index 2 for needs (NEEDS), consisting of the FIN items. The combination of both indices forms the basis for assigning ICs to 4 different risk profiles: currently stable ICs, ICs with unmet needs, psychologically burdened ICs, and high-risk ICs.

a Presentation of the scales is based on Ullrich et al [52], which is published under Creative Commons Attribution 4.0 International License [55].

#### Baseline Questionnaire (t0)

In addition to the scales mentioned, the baseline questionnaire includes questions on sociodemographic characteristics of both the IC and the patient, as well as potential associated factors. Table 2 gives an overview of the included items and scales. All baseline data were collected without planned missing values. The German baseline questionnaire is displayed in Multimedia Appendix 4.

**Table 2.** Included items and scales in the baseline questionnaire for assessing potential associated factors on psychological burden and supportive care needs.

Questionnaire or scale	Description	Analyses
Sociodemographics		
Single items	For example, age and gender of the informal caregiver and the patient.	Single-item analyses.
Socioeconomic status (SES; Winkler Index [56,57])	The Winkler Index is a composite indicator score for assessing the socioeconomic status of a person, taking into account the highest school qualification, highest professional qualification, occupational position, and monthly household net income. Classifications are low, medium, and high SES.	Syntax-based calculation of a score and allocation to a social class: lower class, middle class, and upper class.
Family functioning		
Brief Assessment of General Family Functioning Scale (BAFFS) [58,59]	The BAFFS is the three 3-item ultra-short version of the General Functioning Scale of the Family Assessment Device. Each item is answered on a 4-point Likert scale ranging from 1=strongly agree to 4=strongly disagree.	Calculation and analysis of a sum score (ranging from 3 to 12). Suggested cutoffs are 3‐6=satisfaction with family functioning and 7‐12=distress with family functioning [58].
Preparedness for the role of being an informal caregiver		
Self-developed items, based on the Preparedness for Caregiving Scale [60]	“All in all: How well prepared do you currently feel in your role as an informal caregiver of a loved one with a terminal illness?” This item is answered on a 5-point Likert scale ranging from 1=not good at all to 5=very well. “Is there anything you would like to be better prepared for in your role?” with free-text option.	Single-item analyses.
State of health of the patient and conditions of care		
Self-developed items^a^	Time since the initial diagnosis. Patients’ overall state of health over the last week. Global item, answered on a 7-point Likert scale ranging from 1=very poor to 7=excellent. Patient’s overall quality of life during the last week. Global item, answered on a 7-point Likert scale ranging from 1=very poor to 7=excellent. Previous use of SPC^b^. Previous location of care.	Single-item analyses.

a Self-developed items are displayed in Multimedia Appendix 5.

b SPC: specialist palliative care.

#### Follow-Up Questionnaires (t1-t10) and Implementation of the Planned Missing Data Design

To minimize the burden of the repeated data collection on the ICs, a design with planned missing data—a multiform design—is implemented for all follow-up questionnaires. As summarized by Little and Rhemtulla [31], this design reduces the number of items each participant answers by randomly splitting the total set of items across several forms. The participants are then randomly assigned to one of the forms, answering only the subset of items [31]. Previous research indicates that collecting data with this design leads to similar results compared to complete data designs and can improve validity by reducing participant burden and fatigue, lowering rates of unplanned missing data, and improving efficiency [31,32,61,62]. The multiform design used in this study is based on the work of Rhemtulla and Hancock [32]. A fixed block X consists of 14 items included in every follow-up questionnaire: current care situation of the patient (including an option to report death), all items from the CAREPAL-8, the 1-item analog scale of the DT, a free-text item about recent events that have influenced the burden and needs of the IC, as well as date and mode (with or without assistance) of questionnaire completion. The items from the following scales—GAD-7, PHQ-9, SF-8, OSLO-3, and FIN—are randomly assigned to 6 blocks (A-F), each containing 7‐8 items. The number of blocks was determined as a function of the time that ICs can be expected to spend completing the follow-up questionnaires (approximately 15‐20 minutes) and the number of items. Each follow-up questionnaire includes the fixed block X and 2 different blocks of A-F. Taking all combinations into account, this results in 15 different follow-up questionnaires. Table 3 gives an overview of the questionnaire composition.

**Table 3.** Composition of the follow-up questionnaires.

Block	Items	Follow-up questionnaire number														
		1	2	3	4	5	6	7	8	9	10	11	12	13	14	15
X fixed	For example, current care situation (patient), CAREPAL-8^a^, DT^b^	✓	✓	✓	✓	✓	✓	✓	✓	✓	✓	✓	✓	✓	✓	✓
A randomized	GAD-7^c^ i4^d^; PHQ-9^e^ i2; OSLO-3^f^ i1, i2; FIN^g^ i5, i9, i15, i18	✓	✓	✓	✓	✓										
B randomized	GAD-7 i6; PHQ-9 i7; SF-8^h^ i3*;* OSLO-3 i3; FIN i4, i6, i12, i13	✓					✓	✓	✓	✓						
C randomized	GAD-7 i2; PHQ-9 i6, i9; SF-8 i1, i6; FIN i7, i14, i20		✓				✓				✓	✓	✓			
D randomized	GAD-7 i5; PHQ-9 i3, i5; SF-8 i4, i7; FIN i3, i8, i19			✓				✓			✓			✓	✓	
E randomized	GAD-7 i1, i7; PHQ-9 i1, i4*;* FIN i2, i10, i11*,* i17				✓				✓			✓		✓		✓
F randomized	GAD-7 i3; PHQ-9 i8; SF-8 i2, i5, i8; FIN i1, i16					✓				✓			✓		✓	✓

a CAREPAL-8: 8-Item Screening Tool for Family Caregiver Burden in Palliative Care.

b DT: Distress Thermometer.

c GAD-7: Generalized Anxiety Disorder 7-item scale (items 1-7).

d i: item.

e PHQ-9: Patient Health Questionnaire Depression Module (items 1-9).

f OSLO-3: Oslo Social Support Scale (items 1-3).

g FIN: Family Inventory of Needs (items 1-20).

h SF-8: Short-Form Health Survey (items 1-8).

The questionnaires are randomly distributed at each follow-up measurement point for every IC. The randomization is realized as follows: randomized sets of follow-up questionnaires are prepared for each study center, with each set containing an equal number of all 15 questionnaires (eg, each questionnaire is counted 5 times, resulting in sets of 5*15=75 questionnaires in total). If a study center collects data in more than 1 setting (eg, palliative care ward and specialist palliative home care team), each setting is provided with a separate set of randomized questionnaires to avoid imbalances. For every measurement point, each IC must receive a questionnaire from this predetermined set. Distributing a set of prepared questionnaires for multiple time points to one IC is not allowed to avoid imbalances that may occur if several questionnaires remain unanswered. The version (questionnaire number 1‐15) of the randomly allocated questionnaire is documented for each IC and measurement point. The implementation of the planned missing data design ensures that each follow-up questionnaire contains only half of the total items—29 to 30 instead of all 61—thereby relevantly reducing its length.

### Statistical Analyses

At first, we will conduct a descriptive analysis; we will report mean and SD for continuous variables and absolute and relative frequencies for categorical variables.

#### Longitudinal Assessment of Burdens and Needs

The cohort will be examined to identify groups with similar trajectories using growth mixture modeling (GMM) techniques [63]. More precisely, latent class growth analysis will be used to determine appropriate grouping structures among ICs. We will use the Bayesian information criterion as a relative measure of model performance and derive the most suitable number of trajectories from this. Factors associated with membership in these groups will be identified using a multinomial, multivariable regression model. Because the baseline assessment period was extended from the initially planned 3 days to 7 days, sensitivity analyses will be conducted using the 3-day subsample. As planned missing values are by design missing completely at random, we will use a multiple imputation approach to impute the missing data. Any additional unplanned missing values, potentially not missing completely at random, will be imputed in a further step if necessary. More precisely, first, we impute missing data at baseline on scale level after calculation of scales. In this step, only unplanned missing data exists. We only take this step for the data we use in the GMM. Second, we impute planned missing data on item level, set replaced unplanned missing data to missing data again, and then, calculate the scales and impute the unplanned missing data on scale level. The imputation model consists of all baseline and follow-up variables. Any missing values resulting from the patient’s death will be set to missing data again. We will use a joint modeling approach with a Weibull distribution of event times to account for missing data due to the patient’s death.

#### Validation of the CAREPAL-8 Screening Tool

The data used to evaluate the psychometric properties of the CAREPAL-8 will be collected completely without planned missing values. To assess convergent validity, Pearson correlation analyses between index 1 and the scores of the GAD-7, PHQ-9, and both component scores of the SF-8, and between index 2 and the FIN will be conducted using baseline data. We expect high correlations of at least |*r*|=0.50. In our first version of the study protocol in 2022, we originally expected at least |*r*|=0.70. After further methodological considerations and before data analyses, we adjusted this value. According to the recommendations of Cohen [64], both values are interpreted as high. Sensitivity to change will be evaluated by using the patient’s care setting during the longitudinal observation as an external criterion, categorizing ICs in 2 groups: those who experienced at least 1 transition between outpatient and inpatient care (or vice versa) and those who did not transition. Clinically, it can be assumed that these transitions occur in response to significant changes in the patient’s health status or in the capacities of the ICs (eg, decompensation of home care due to IC overload) and are reflected in changes in the indices. We will evaluate whether a minimally important difference of 0.5 SD exists between these groups for each index. As an additional criterion, we will analyze the free-text item about recent events that have changed the burden and needs of the IC. The longitudinal data used in these analyses are part of the fixed block X, which is implemented in every questionnaire version. Supplementary analyses will assess the sensitivity and specificity of the instrument by determining cutoffs based on receiver operating characteristic analyses for both indices (index 1: PSYQOL and index 2: NEEDS). From this, we will analyze which cutoff for each index best predicts a change in care setting (transition vs no-transition group).

Statistical analyses will be conducted using the statistical software packages R (version 4.1.2 or newer; R Foundation for Statistical Computing) and SPSS Statistics (version 29 or newer; IBM Corp).

### Sample Size Calculation

To determine the required sample size for the study, power calculations were conducted using PASS 15.0.3, module “One-Way Analysis of Variance F-Tests using Effect Size.”

#### Longitudinal Assessment of Burdens and Needs

As the number of trajectory types is an outcome of this exploratory study, sample size calculation for GMMs is not feasible due to the uncertainty of key parameters. As a proxy, a mean comparison of the baseline variables between trajectory types is used. An IC is part of the analysis population if the baseline data and at least 1 follow-up questionnaire are valid. Various scenarios with 3 to 6 trajectory types were calculated.

Assuming, for reasons of clinical practicability, a highest number of 6 trajectory types and a small effect size (η^2^=0.025), we would need 85 ICs per group (total of 510 ICs) to detect this effect size with a power of 80% and a 2-sided type I error of 5%. This calculation assumes equal group sizes.

Sijbrandij et al [65] examined cases where estimation or performance issues arise when using GMMs. The authors concluded that no performance issues occur with sample sizes of 1000 individuals, while sample sizes of 300 individuals can present performance challenges, which can only be addressed by limiting variance parameters. Furthermore, we cite the study of Kim [66], which investigates GMMs in the sample size range targeted in this study. Additionally, we searched for studies using GMMs [67-72], which included sample sizes ranging from 129 to 6987 individuals. Our targeted sample size of 510 ICs is on the lower end of this range, but it is similar to another study conducted in the palliative care setting [71].

#### Second Study Objective: Validation of the CAREPAL-8 Screening Tool

To calculate the required sample size for assessing convergent validity, we assume a Pearson correlation coefficient of 0.50 and a 2-sided 95% CI with a width of 0.20. Based on these parameters, a sample of 219 ICs is required. Therefore, by the end of the study, we aim to have baseline data from 510 ICs, along with at least 1 additional follow-up assessment, to demonstrate the described effects and apply the described models.

## Results

The funding period of this 3-year study started on March 15, 2023. Data collection commenced on July 31, 2023, at 3 study centers, with the remaining 13 centers starting successively from November 2023. Recruitment of new participants ended on December 31, 2024. After completing all follow-up measurement points, we will conclude data entry and start data cleaning. We expect to evaluate the cleaned and complete dataset from July 2025, and results are expected from March 2026. The protocol does not include any interim analyses. The data will be published in peer-reviewed journals, with the publication process expected to commence in summer 2026.

## Discussion

The objectives of this cohort study are twofold: first, to gain insights into the trajectories of psychosocial burden and supportive care needs of ICs in the course of SPC, and second, to validate the newly developed multidimensional screening tool CAREPAL-8 that enables classification of ICs according to different risk profiles with consecutive recommendations for clinical care. Therefore, we are implementing a prospective, longitudinal, multicenter design with up to 11 measurement points.

### Potential Obstacles and Proposed Solutions

Various limitations and obstacles are conceivable in the course of this study.

#### Recruitment of ICs and Data Collection

To apply the statistical models mentioned, baseline data plus at least 1 additional follow-up questionnaire from 510 ICs are required. Previous studies with ICs and similar inclusion and exclusion criteria have shown that baseline data could be collected from ICs of 30%‐40% of patients admitted to specialist inpatient palliative care [34,52]. Given the weekly follow-up assessments, a high degree of attrition must be anticipated in this study, primarily due to the rapid onset of patient death following admission to SPC or the study withdrawal of the IC. Consequently, we plan to recruit more ICs to account for this attrition. To facilitate study enrollment, we focus on the direct recruitment of ICs by trained study staff at the patient’s location of care. Flyers and other promotional materials for the study are available to support this process. To maintain the motivation of the ICs throughout the study, several measures are determined. First, all ICs are informed in a consistent and thorough manner about the study procedures, including the background, aims, and specifics related to the planned missing data design (eg, randomized order of items from different scales). Second, all study materials are carefully prepared and include contact information for any questions about the study as well as the psychological consultation hour. To facilitate the study processes for ICs, flexible options of receiving and transmitting the questionnaires are being offered: questionnaires can be distributed and returned at the participating study center or patient care location, sent by mail, or completed during a phone call. If questionnaires are not returned, study staff will follow up with ICs. The return rates of questionnaires as well as the overall data collection process are closely monitored so that potential difficulties and obstacles can be identified and addressed early on.

#### Multicenter Collaboration and Inclusion of Different Care Settings

Other challenges may occur regarding the collaboration across 16 study centers in Germany. To ensure high data quality and comparability, a standardized and field-tested training for data collection and quality control is conducted at all study centers prior to data collection. Regular project meetings are held during the data collection period, and additional consultations are available at any time during the study process to address individual conditions. Due to the different conditions and resources across study centers, various ways for exchanging relevant study information between the centers and the leading center are implemented. For study staff without regular computer access (eg, a nurse in a specialist palliative home care team), relevant information can be documented on prepared paper forms and transmitted via fax or mail. Members of the study team who primarily work on computers (eg, scientific personnel) can use prepared Microsoft Excel sheets and share pseudonymized information via password-protected cloud systems. Quarterly newsletters are sent to inform study staff about past, present, and upcoming project activities, as well as recruitment progress and sample size. An expense allowance of US $319.2 (€280) is granted to each study center for every IC enrolled in the study with a valid baseline questionnaire.

With regard to the different SPC settings, recruitment strategies may differ. For example, the recruitment of ICs of patients who are cared for by multiprofessional SPC teams in hospitals might face challenges, for example, due to not meeting the ICs because of limited visiting hours in the hospital. We therefore developed different materials and recruitment procedures such as cover letters for ICs that can be deposited in the hospital room of the patient. If the patient transits between settings during the study period, data collection procedures might be adapted, for example, sending the questionnaire via mail instead of depositing the questionnaire at the location of care.

The multicenter design of the study, while essential for broad data collection and generalizability, presents potential challenges in ensuring transparent and equitable attribution of contributions across multiple institutions. To address this, authorship of resulting publications will be determined in accordance with the criteria established by the International Committee of Medical Journal Editors and the Contributor Role Taxonomy, ensuring that all contributions are appropriately recognized and documented.

#### Implementation of the Planned Missing Data Design

The implementation of the planned missing data design involves several logistical and methodological challenges. Due to the random allocation of all items across 15 questionnaire versions, the preparation of the questionnaires required careful quality control to minimize the risk of errors. To address this, sufficient time was allocated for questionnaire preparation, and independent checks were performed by different members of the study team.

Particular attention is given to ensuring that the questionnaire layout is clear and does not increase confusion among ICs. Due to the random allocation of items, the thematic relationship between questions is not always apparent to participants, which may potentially lead to a perceived lack of coherence. To address this issue, we grouped items from the same questionnaires together and numbered items consecutively. All study-related information is presented in a nonintrusive manner, for example, by displaying the questionnaire version in a small font size in the lower right corner of each page.

All study staff are trained in the specific features of the planned missing data design and will provide ICs with appropriate instructions when distributing questionnaires. Randomization is centrally performed by the leading center as described in the Follow-Up Questionnaires (t1-t10) and Implementation of the Planned Missing Data Design section. As a result, each study center receives prerandomized questionnaire sets and is instructed to distribute the next available questionnaire within the set.

The design also requires specific documentation procedures. The questionnaire version administered to each IC at each measurement point is documented using structured paper- and electronic forms. Data entry is complex due to the existence of 15 questionnaire versions per measurement point, resulting in a structured and extensive data matrix. To ensure data quality, all personnel involved in data entry receive prior training, and double data entry procedures are implemented. This study is planned, conducted, and analyzed by an experienced multiprofessional research team, including experts in medical biometry.

### Conclusions

With this cohort study, we aim to collect data on the psychosocial burden and supportive care needs of ICs in the course of SPC. We anticipate gaining insights into trajectories of burdens and needs, as well as factors associated with these trajectories. If the CAREPAL-8 proves to be valid, a brief tool would be available for classifying ICs based on different risk profiles of multidimensional caregiver burden, with specific recommendations for daily practice. Additionally, if the implementation of the planned missing data design proves to be advantageous both for the ICs as well as data quality, this study could serve as a model for further studies with vulnerable populations. Ultimately, we expect that the findings will have significant implications for palliative care providers and policymakers, helping them better recognize and address the unique challenges faced by ICs in everyday palliative care.

## Supplementary material

10.2196/78076 Multimedia Appendix 1 Adapted version of the SPIRIT (Standard Protocol Items: Recommendations for Interventional Trials) schedule.

10.2196/78076 Multimedia Appendix 2 Items from the World Health Organization Trial Registration Data Set.

10.2196/78076 Multimedia Appendix 3 Overview of all study centers and settings.

10.2196/78076 Multimedia Appendix 4 German version of the baseline questionnaire.

10.2196/78076 Multimedia Appendix 5 Self-developed items used in the baseline questionnaire.

10.2196/78076 Checklist 1 SPIRIT checklist.

## Abbreviations

CAREPAL-8: 8-Item Screening Tool for Family Caregiver Burden in Palliative Care
DT: Distress Thermometer
FIN: Family Inventory of Needs
GAD-7: Generalized Anxiety Disorder 7-item scale
GMM: growth mixture modeling
IC: informal caregiver
OSLO-3: Oslo Social Support Scale
PHQ-9: Patient Health Questionnaire Depression Module
PSYQOL: psychopathological symptoms and quality of life
SF-8: Short-Form Health Survey
SPC: specialist palliative care
SPIRIT: Standard Protocol Items: Recommendations for Interventional Trials

## References

[1] Alam S, Hannon B, Zimmermann C (2020). Palliative care for family caregivers. J Clin Oncol.

[2] (2020). Palliativmedizin für Patienten mit einer nicht-heilbaren Krebserkrankung, Langversion 2.2, AWMF-Registernummer: 128/001OL. Leitlinienprogramm Onkologie (Deutsche Krebsgesellschaft, Deutsche Krebshilfe, AWMF).

[3] Choi S, Seo J (2019). Analysis of caregiver burden in palliative care: an integrated review. Nurs Forum.

[4] Wang T, Molassiotis A, Chung BPM, Tan JY (2018). Unmet care needs of advanced cancer patients and their informal caregivers: a systematic review. BMC Palliat Care.

[5] Ullrich A, Marx G, Bergelt C (2021). Supportive care needs and service use during palliative care in family caregivers of patients with advanced cancer: a prospective longitudinal study. Support Care Cancer.

[6] Hashemi M, Irajpour A, Taleghani F (2018). Caregivers needing care: the unmet needs of the family caregivers of end-of-life cancer patients. Support Care Cancer.

[7] Hudson P, Payne S (2011). Family caregivers and palliative care: current status and agenda for the future. J Palliat Med.

[8] Dumont S, Fillion L, Gagnon P, Bernier N (2008). A new tool to assess family caregivers’ burden during end-of-life care. J Palliat Care.

[9] Zwahlen D, Hagenbuch N, Carley MI, Recklitis CJ, Buchi S (2008). Screening cancer patients’ families with the distress thermometer (DT): a validation study. Psychooncology.

[10] Kroenke K, Spitzer RL, Williams JB (2001). The PHQ-9: validity of a brief depression severity measure. J Gen Intern Med.

[11] Kocalevent RD, Hinz A, Brähler E (2013). Standardization of the depression screener patient health questionnaire (PHQ-9) in the general population. Gen Hosp Psychiatry.

[12] Ullrich A, Bergelt C, Marx G (2024). The CAREPAL-8: a short screening tool for multidimensional family caregiver burden in palliative care. BMC Palliat Care.

[13] Hudson PL, Trauer T, Graham S (2010). A systematic review of instruments related to family caregivers of palliative care patients. Palliat Med.

[14] Given CW, Given B, Stommel M, Collins C, King S, Franklin S (1992). The caregiver reaction assessment (CRA) for caregivers to persons with chronic physical and mental impairments. Res Nurs Health.

[15] Bee PE, Barnes P, Luker KA (2009). A systematic review of informal caregivers’ needs in providing home-based end-of-life care to people with cancer. J Clin Nurs.

[16] Hart NH, Crawford-Williams F, Crichton M (2022). Unmet supportive care needs of people with advanced cancer and their caregivers: a systematic scoping review. Crit Rev Oncol Hematol.

[17] Williams AL, McCorkle R (2011). Cancer family caregivers during the palliative, hospice, and bereavement phases: a review of the descriptive psychosocial literature. Palliat Support Care.

[18] See Jia Wen F, Teo Eng Ai I, Malhotra C, COMPASS Study Group (2022). Longitudinal trajectories of caregiving experiences among primary informal caregivers of patients with metastatic solid cancer (stage IV). Psychooncology.

[19] Guerriere D, Husain A, Zagorski B (2016). Predictors of caregiver burden across the home-based palliative care trajectory in Ontario, Canada. Health Soc Care Community.

[20] Genderson MW, Thomson MD, Siminoff LA (2024). Where you begin is not necessarily where you end: the mental and physical health trajectories of cancer caregivers over time. Support Care Cancer.

[21] Bijnsdorp FM, Onwuteaka-Philipsen BD, Boot CRL, van der Beek AJ, Pasman HRW (2022). Caregiver’s burden at the end of life of their loved one: insights from a longitudinal qualitative study among working family caregivers. BMC Palliat Care.

[22] Grunfeld E, Coyle D, Whelan T (2004). Family caregiver burden: results of a longitudinal study of breast cancer patients and their principal caregivers. CMAJ.

[23] Lee KC, Chang WC, Chou WC (2013). Longitudinal changes and predictors of caregiving burden while providing end-of-life care for terminally ill cancer patients. J Palliat Med.

[24] Shield T, Bayliss K, Hodkinson A (2023). What factors are associated with informal carers’ psychological morbidity during end-of-life home care? A systematic review and thematic synthesis of observational quantitative studies. Health Soc Care Deliv Res.

[25] Tallman K, Greenwald R, Reidenouer A, Pantel L (2012). Living with advanced illness: longitudinal study of patient, family, and caregiver needs. Perm J.

[26] Nysaeter TM, Olsson C, Sandsdalen T, Hov R, Larsson M (2024). Family caregivers’ preferences for support when caring for a family member with cancer in late palliative phase who wish to die at home—a grounded theory study. BMC Palliat Care.

[27] DuBenske LL, Wen KY, Gustafson DH (2008). Caregivers’ differing needs across key experiences of the advanced cancer disease trajectory. Palliat Support Care.

[28] Pop RS, Mosoiu DV, Puia A, Tint D (2023). Comparison of the burden evolution of the family caregivers for patients with cancer and nononcological diseases who need palliative care: a prospective longitudinal study. Palliat Med Rep.

[29] Tanco K, Prado B, Qian Y (2021). A comparison of caregiver burden of patients with advanced cancer in different palliative cancer care settings. J Palliat Med.

[30] Chan AW, Tetzlaff JM, Altman DG (2013). SPIRIT 2013 statement: defining standard protocol items for clinical trials. Ann Intern Med.

[31] Little TD, Rhemtulla M (2013). Planned missing data designs for developmental researchers. Child Dev Perspect.

[32] Rhemtulla M, Hancock GR (2016). Planned missing data designs in educational psychology research. Educ Psychol.

[33] Wikert J, Gesell D, Bausewein C (2022). Specialist palliative care classification: typology development. BMJ Support Palliat Care.

[34] Oechsle K, Ullrich A, Marx G (2019). Psychological burden in family caregivers of patients with advanced cancer at initiation of specialist inpatient palliative care. BMC Palliat Care.

[35] Ullrich A, Bokemeyer C, Oechsle K (2024). Standardized training for recruitment of informal caregivers in palliative care research. Oncol Res Treat.

[36] LeBlanc TW, Lodato JE, Currow DC, Abernethy AP (2013). Overcoming recruitment challenges in palliative care clinical trials. J Oncol Pract.

[37] Higginson IJ, Evans CJ, Grande G (2013). Evaluating complex interventions in end of life care: the MORECare statement on good practice generated by a synthesis of transparent expert consultations and systematic reviews. BMC Med.

[38] Ullrich A, Schulz H, Goldbach S (2021). Need for additional professional psychosocial and spiritual support in patients with advanced diseases in the course of specialist palliative care—a longitudinal observational study. BMC Palliat Care.

[39] Just J, Schmitz MT, Grabenhorst U (2021). Factors influencing length of survival in ambulatory palliative care—a cross sectional study based on secondary data. BMC Palliat Care.

[40] Gothe H, Brinkmann C, Schmedt N, Walker J, Ohlmeier C (2022). Is there an unmet medical need for palliative care services in Germany? Incidence, prevalence, and 1-year all-cause mortality of palliative care sensitive conditions: real-world evidence based on German claims data. J Public Health (Berl).

[41] National Comprehensive Cancer Network (2003). Distress management. Clinical practice guidelines. J Natl Compr Canc Netw.

[42] Mehnert A, Müller D, Lehmann C, Koch U (2006). The German version of the NCCN Distress Thermometer: validation of a screening instrument for assessment of psychosocial distress in cancer patients [Article in German]. Zeitschrift für Psychiatrie, Psychologie und Psychotherapie.

[43] Spitzer RL, Kroenke K, Williams JBW, Löwe B (2006). A brief measure for assessing generalized anxiety disorder: the GAD-7. Arch Intern Med.

[44] Löwe B, Decker O, Müller S (2008). Validation and standardization of the Generalized Anxiety Disorder Screener (GAD-7) in the general population. Med Care.

[45] Löwe B, Spitzer RL, Gräfe K (2004). Comparative validity of three screening questionnaires for DSM-IV depressive disorders and physicians’ diagnoses. J Affect Disord.

[46] Ware Jr JE, Kosinski M, Dewey JE, Gandek B (2001). How to Score and Interpret Single-Item Health Status Measures: A Manual for Users of the SF-8 Health Survey.

[47] Beierlein V, Morfeld M, Bergelt C, Bullinger M, Brähler E (2012). Measuring health-related quality of life with the SF-8: German norms from a representative self-administered survey [Article in German]. Diagnostica.

[48] Kristjanson LJ, Atwood J, Degner LF (1995). Validity and reliability of the family inventory of needs (FIN): measuring the care needs of families of advanced cancer patients. J Nurs Meas.

[49] Schur S, Neubauer M, Amering M (2015). Validation of the family inventory of needs (FIN) for family caregivers in palliative care. Palliat Support Care.

[50] Meltzer H, Nosikov A, Gudex C (2003). EUROHIS: Developing Common Instruments for Health Surveys.

[51] Kocalevent RD, Berg L, Beutel ME (2018). Social support in the general population: standardization of the Oslo Social Support Scale (OSSS-3). BMC Psychol.

[52] Ullrich A, Ascherfeld L, Marx G, Bokemeyer C, Bergelt C, Oechsle K (2017). Quality of life, psychological burden, needs, and satisfaction during specialized inpatient palliative care in family caregivers of advanced cancer patients. BMC Palliat Care.

[53] Bužgová R, Kozáková R (2016). Psychometric evaluation of a Czech version of the family inventory of needs. Palliat Support Care.

[54] Bøen H, Dalgard OS, Bjertness E (2012). The importance of social support in the associations between psychological distress and somatic health problems and socio-economic factors among older adults living at home: a cross sectional study. BMC Geriatr.

[55] Attribution 4.0 international (CC BY 4.0). Creative Commons.

[56] Winkler J, Ahrens W, Bellach BM, Jöckel KH (1998). Measurement of Sociodemographic Characteristics in Epidemiology [Book in German].

[57] Winkler J, Stolzenberg H (2009). Adjustierung Des Sozialen-Schicht-Index Für Die Anwendung Im Kinder- Und Jugendgesundheitssurvey (KiGGS) [Book in German].

[58] Mansfield AK, Keitner GI, Sheeran T (2019). The Brief Assessment of Family Functioning Scale (BAFFS): a three-item version of the general functioning scale of the family assessment device. Psychother Res.

[59] Spitzer C, Lübke L, Göbel P (2022). Assessment of general family functioning: psychometric evaluation of the German version of the Brief Assessment of Family Functioning Scale [Article in German]. Psychother Psychosom Med Psychol.

[60] Archbold PG, Stewart BJ, Greenlick MR, Harvath T (1990). Mutuality and preparedness as predictors of caregiver role strain. Res Nurs Health.

[61] Wu W, Jia F (2021). Applying planned missingness designs to longitudinal panel studies in developmental science: an overview. New Dir Child Adolesc Dev.

[62] Smits N, Vorst HCM (2007). Reducing the length of questionnaires through structurally incomplete designs: an illustration. Learn Individ Differ.

[63] Muthén B, Kaplan D (2004). The SAGE Handbook of Quantitative Methodology for the Social Sciences.

[64] Cohen J (1988). Statistical Power Analysis for the Behavioral Sciences.

[65] Sijbrandij JJ, Hoekstra T, Almansa J, Peeters M, Bültmann U, Reijneveld SA (2020). Variance constraints strongly influenced model performance in growth mixture modeling: a simulation and empirical study. BMC Med Res Methodol.

[66] Kim SY (2012). Sample size requirements in single- and multiphase growth mixture models: a Monte Carlo simulation study. Struct Equ Model.

[67] Melchior H, Schulz H, Kriston L (2016). Symptom change trajectories during inpatient psychotherapy in routine care and their associations with long-term outcomes. Psychiatry Res.

[68] Ames ME, Wintre MG (2016). Growth mixture modeling of adolescent body mass index development: longitudinal patterns of internalizing symptoms and physical activity. J Res Adolesc.

[69] Gilthorpe MS, Dahly DL, Tu YK, Kubzansky LD, Goodman E (2014). Challenges in modelling the random structure correctly in growth mixture models and the impact this has on model mixtures. J Dev Orig Health Dis.

[70] Infurna FJ, Grimm KJ (2017). The use of growth mixture modeling for studying resilience to major life stressors in adulthood and old age: lessons for class size and identification and model selection. J Gerontol B Psychol Sci Soc Sci.

[71] Lyon ME, Caceres S, Scott RK (2021). Advance care planning-complex and working: longitudinal trajectory of congruence in end-of-life treatment preferences: an RCT. Am J Hosp Palliat Care.

[72] Kristensen P, Dyregrov K, Gjestad R (2020). Different trajectories of prolonged grief in bereaved family members after terror. Front Psychiatry.

[73] Suchikova Y, Tsybuliak N, Teixeira da Silva JA, Nazarovets S (2026). GAIDeT (Generative AI Delegation Taxonomy): a taxonomy for humans to delegate tasks to generative artificial intelligence in scientific research and publishing. Account Res.

