# Supplementary material for: Psychosocial Burden and Supportive Care Needs of Informal Caregivers in Specialist Palliative Care: Protocol of a Multicenter Longitudinal Cohort Study to Identify Trajectories and Validate the Multidimensional Screening Tool CAREPAL-8
Source: JMIR Res Protoc. 2026 Jul 31;15:e78076. doi: 10.2196/78076 (PMC13427073; doi:10.2196/78076)
Supplement: Multimedia Appendix 1 [file resprot-v15-e78076-s001.pdf]

Adapted version of the SPIRIT schedule.

	**STUDY PERIOD**											
	**Enrollment**	**Baseline**	**Follow-up (starting from the date of written informed consent)**									
**TIMEPOINT**	**Admission:** Written informed consent within 3 days, max. within 7 days	t_0 Within 3 days, max. within 7 days after admission	t_1 +7 days	t_2 +7 days	t_3 +7 days	t_4 +7 days	t_5 +7 days	t_6 +7 days	t_7 +7 days	t_8 +7 days	t_9 +7 days	t_{10} +7 days
**ENROLLMENT:**												
Eligibility screen	X											
Written informed consent	X											
**ASSESSMENTS:**												
Outcome variables:												
CAREPAL-8		X	X	X	X	X	X	X	X	X	X	X
DT		X	X	X	X	X	X	X	X	X	X	X
GAD-7		X	X[1]	X[1]	X[1]	X[1]	X[1]	X[1]	X[1]	X[1]	X[1]	X[1]
PHQ-9		X	X[1]	X[1]	X[1]	X[1]	X[1]	X[1]	X[1]	X[1]	X[1]	X[1]
OSLO-3		X	X[1]	X[1]	X[1]	X[1]	X[1]	X[1]	X[1]	X[1]	X[1]	X[1]
SF-8		X	X[1]	X[1]	X[1]	X[1]	X[1]	X[1]	X[1]	X[1]	X[1]	X[1]
FIN		X	X[1]	X[1]	X[1]	X[1]	X[1]	X[1]	X[1]	X[1]	X[1]	X[1]
Other main variables:												
Socio-demo-graphic variables		X										
BAFFS		X										
Preparedness for the role of being an informal caregiver		X										
State of health of the patient		X										
Conditions of care of the patient		X	X	X	X	X	X	X	X	X	X	X

Rows for interventions and columns for allocation and close-out are not shown as this is not an interventional study. [1]Items of these scales are randomized (planned missing data design). CAREPAL-8: 8-item Screening Tool for Family Caregiver Burden in Palliative Care; DT: Distress Thermometer; GAD-7: Generalized Anxiety Disorder Scale; PHQ-9: Patient-Health-Questionnaire depression module; OSLO-3: Oslo Social Support Scale; SF-8: Short-Form Health Survey; FIN: Family Inventory of Needs; BAFFS: Brief Assessment of General Family Functioning Scale.
