# Supplementary material for: Psychosocial Burden and Supportive Care Needs of Informal Caregivers in Specialist Palliative Care: Protocol of a Multicenter Longitudinal Cohort Study to Identify Trajectories and Validate the Multidimensional Screening Tool CAREPAL-8
Source: JMIR Res Protoc. 2026 Jul 31;15:e78076. doi: 10.2196/78076 (PMC13427073; doi:10.2196/78076)
Supplement: Multimedia Appendix 2 [file resprot-v15-e78076-s002.pdf]

Items from the World Health Organization Trial Registration Data Set.

Primary registry and trial identifying number	German Clinical Trial Register (DRKS00030480).
Date of registration in primary registry	19.01.2023
Secondary identifying numbers	German Cancer Aid: 70115093<br>Ethics committee of the Medical Association of Hamburg: 2022-100878-BO-ff<br>Ethics committees of Dresden University of Technology: SR+BO-EK-388092023<br>Ethics Committee at the Faculty of Medicine of the Heinrich Heine University Düsseldorf: 2022-2210-Klinische Forschung<br>Ethics Committee of the Friedrich Alexander University Erlangen-Nürnberg: 23-82-Bn<br>Ethics Committee at the Faculty of Medicine of the Friedrich Schiller University Jena: 2022-2802-Bef<br>Ethics Committee at the Faculty of Medicine at Christian Albrechts University in Kiel: B 244/24<br>Ethics Committee of Hannover Medical School: 10815_BO_K_2023<br>Medical Ethics Committee at Julius Maximilian University of Würzburg: 33/24_z-am
Source(s) of monetary or material support	German Cancer Aid
Primary sponsor	University Medical Center Hamburg-Eppendorf
Secondary sponsor(s)	N/A
Contact for public queries	CH: c.haufschild@uke.de , AU: a.ullrich@uke.de
Contact for scientific queries	CH: c.haufschild@uke.de , AU: a.ullrich@uke.de
Public title	Protocol of a longitudinal study on burden and needs of informal caregivers in palliative care
Scientific title	Psychosocial burden and supportive care needs of informal caregivers in specialist palliative care: Protocol of a multicenter longitudinal cohort study to identify trajectories and validate the multidimensional screening tool CAREPAL-8
Countries of recruitment	Germany
Health condition(s) or problem(s) studied	Psychosocial burden, unmet supportive care needs (informal caregivers of patients receiving specialist palliative care)
Intervention(s)	N/A
Key inclusion and exclusion criteria	Inclusion criteria: being a close relative or a significant person in the patient's life who provides unpaid care or support to a patient with an advanced incurable disease, age ≥ 18 years. Exclusion criteria: patient's imminent death, legal

	guardianship without a personal connection to the patient, and insufficient German language skills of the informal caregiver
Study type	Cohort study
Date of first enrollment	31.07.2023
Target sample size	510
Recruitment status	Recruitment completed, data collection ongoing
Primary outcome(s)	Multidimensional caregiver burden: CAREPAL-8<br>Distress: DT<br>Anxiety: GAD-7<br>Depression: PHQ-9<br>Health-related quality of life: SF-8<br>Unmet needs: FIN<br>Social support: OSLO-3
Key secondary outcomes	Factors potentially associated with the psychosocial burden and unmet supportive care needs of informal caregivers
Ethics Review	Status: Approved (primary and secondary ethical approvals)<br>Name and contact details ethics committee (primary approval):<br>Ethics committee of the Medical Association of Hamburg<br>Weidestraße 122 b<br>22083 Hamburg<br>E-Mail: ethik@aekhh.de<br>Identifier of primary approval: 2022-100878-BO-ff<br>Date of primary approval: 26.07.2022
Completion date	To be determined
Summary Results	To be determined
IPD sharing statement	Plan to share IPD: No
