# Supplementary material for: Psychosocial Burden and Supportive Care Needs of Informal Caregivers in Specialist Palliative Care: Protocol of a Multicenter Longitudinal Cohort Study to Identify Trajectories and Validate the Multidimensional Screening Tool CAREPAL-8
Source: JMIR Res Protoc. 2026 Jul 31;15:e78076. doi: 10.2196/78076 (PMC13427073; doi:10.2196/78076)
Supplement: Multimedia Appendix 3 [file resprot-v15-e78076-s003.pdf]

Overview of all study centers and settings.

Nr.	Federal state of Germany	Institution, department, city/region	Academic/non-academic facility	Included SPC settings
1	Hamburg	Universitätsklinikum Hamburg-Eppendorf, II. Medizinische Klinik, Palliativmedizin, Hamburg (leading study center)	Academic	Palliative care ward<br>Hospital-based multi-professional SPC team
2	Hamburg	Agaplesion Diakonieklinikum Hamburg, Hamburg	Non-academic	Palliative care ward
3	Hamburg	Asklepios Westklinikum Hamburg, Hamburg	Non-academic	Palliative care ward
4	Hamburg	Das Palliativteam Hamburg, Hamburg	Non-academic	Specialist palliative home care team
5	Hamburg	Palliative Care Team Süderelbe, Hamburg	Non-academic	Specialist palliative home care team
6	Hamburg	Palliativnetz Hamburg-West, Hamburg	Non-academic	Specialist palliative home care team
7	Hamburg	PalliativPartner Hamburg, Hamburg	Non-academic	Specialist palliative home care team
8	Schleswig-Holstein	Universitätsklinikum Schleswig-Holstein (Campus Kiel), Interdisziplinäres Zentrum für Schmerz- und Palliativmedizin/ Klinik für Strahlentherapie/ Klinik für Innere Medizin II mit den Schwerpunkten Hämatologie und Onkologie, Kiel	Academic	Palliative care ward<br>Hospital-based multi-professional SPC team
9	Schleswig-Holstein	Palliativnetz Travebogen, Lübeck	Non-academic	Specialist palliative home care team
10	Lower Saxony	Medizinische Hochschule Hannover, Institut für Allgemeinmedizin und Palliativmedizin, Hannover	Academic	Palliative care ward
11	North Rhine-Westphalia	Universitätsklinikum Düsseldorf, Interdisziplinäres Zentrum für Palliativmedizin, Düsseldorf	Academic	Palliative care ward<br>Hospital-based multi-professional SPC team<br>Specialist palliative home care team
12	Saxony	Universitätsklinikum Dresden, UniversitätsPalliativCentrum, Dresden	Academic	Palliative care ward

[continued]				
**Nr.**	**Federal state of Germany**	**Institution, department, city/region**	**Academic/non-academic facility**	**Included SPC settings**
13	Thuringia	Universitätsklinikum Jena, Klinik für Innere Medizin II, Abteilung Palliativmedizin, Jena	Academic	Palliative care ward<br>Hospital-based multi-professional SPC team<br>Specialist palliative home care team
14	Bavaria	Universitätsklinikum Erlangen, Palliativmedizinische Abteilung, Erlangen	Academic	Palliative care ward
15	Bavaria	Universitätsklinikum Regensburg, Zentrum für Palliativmedizin, Regensburg	Academic	Palliative care ward<br>Specialist palliative home care team
16	Bavaria	Universitätsklinikum Würzburg, Interdisziplinäres Zentrum Palliativmedizin, Würzburg	Academic	Palliative care ward<br>Hospital-based multi-professional SPC team

SPC: specialist palliative care
