# Supplementary material for: Psychosocial Burden and Supportive Care Needs of Informal Caregivers in Specialist Palliative Care: Protocol of a Multicenter Longitudinal Cohort Study to Identify Trajectories and Validate the Multidimensional Screening Tool CAREPAL-8
Source: JMIR Res Protoc. 2026 Jul 31;15:e78076. doi: 10.2196/78076 (PMC13427073; doi:10.2196/78076)
Supplement: Multimedia Appendix 4 [file resprot-v15-e78076-s004.pdf]

Vom Personal auszufüllen:

Code-Nr.: ______________

Ausgabe- bzw. Versanddatum:

Tag		Monat		Jahr	

Belastungen und Bedürfnisse von Angehörigen in der Palliativversorgung

Fragebogen für Angehörige

Erstbefragung

Sehr geehrte:r Angehörige:r,

die unheilbare Erkrankung eines nahestehenden Menschen bringt für die **Angehörigen, d.h. für die Familie, Verwandten und Freund:innen**, häufig vielfältige Belastungen mit sich. Gleichzeitig finden die Bedürfnisse von Angehörigen in der palliativen Erkrankungssituation des/der Patient:in nicht immer ausreichend Berücksichtigung.

Unser Ziel ist es, die Betreuung von Patient:innen und Angehörigen stetig weiter zu verbessern. Es ist daher sehr wichtig für uns zu wissen, was **Ihre Belastungen** als Angehörige:r sind und wo **Sie** sich **Beratung und Unterstützung** wünschen. Um dies zu erfahren, möchten wir Sie herzlich bitten, sich etwa **45 Minuten** Zeit zu nehmen und diesen Fragebogen zu beantworten. Vielen Dank!

Die Beantwortung der Fragen ist **freiwillig**. Für die wissenschaftliche Auswertung ist es aber sehr wichtig, **vollständige Angaben zu erhalten**. Daher möchten wir Sie bitten, alle Fragen zu lesen und zu beantworten. Dabei gibt es keine „richtigen“ oder „falschen“ Antworten. Wenn Sie einmal nicht sicher sind, wählen Sie bitte die Antwort, die spontan am ehesten zutrifft. **Es kann vorkommen, dass sich manche Fragen sehr ähneln** oder **wiederholt vorkommen.** Dies ist aus wissenschaftlichen Gründen nicht anders möglich und wir bitten dies zu entschuldigen.

Beispielfrage

	Wie wichtig ist Ihnen das Bedürfnis derzeit?						Wird das Bedürfnis derzeit erfüllt?		
**Ich habe das Bedürfnis, …**	**nicht wichtig**	**etwas wichtig**	**mittel wichtig**	**sehr wichtig**	**extrem wichtig**		**erfüllt**	**teil-weise erfüllt**	**nicht erfüllt**
1. dass meine Fragen ehrlich beantwortet werden	❑	☒	❑	❑	❑	➔	☒	❑	❑

Wenn Ihnen dieses Bedürfnis **zum Beispiel** etwas wichtig ist, kreuzen Sie das entsprechende Kästchen an.

Wenn dieses Bedürfnis **zum Beispiel** erfüllt ist, kreuzen Sie das entsprechende Kästchen an.

An einigen Stellen werden Sie darum gebeten, handschriftlich Angaben zu machen. Dies ist dann mit dem folgenden Symbol gekennzeichnet: ✎.

Wenn Sie Fragen zur Studie haben, können Sie sich direkt an [redacted] wenden, die für die Koordination der Studie verantwortlich ist. Uns ist bewusst, dass in Anbetracht dessen, was Sie derzeit erleben, einige Fragen für Sie schwierig sein könnten. Falls Sie durch das Beantworten des Fragebogens spüren, dass Sie sich Unterstützung wünschen, können Sie sich bei dem betreuenden Palliativteam oder gerne auch bei [redacted] melden.

[redacted]
Wissenschaftliche Mitarbeiterin, Psychologin
Studienkoordinatorin und Ansprechpartnerin

Palliativmedizin, II. Medizinische Klinik
Zentrum für Onkologie
Universitätsklinikum Hamburg-Eppendorf

Sprechzeiten
montags, 15:00 – 16:00 Uhr
freitags, 10:00 – 11:00 Uhr

☎ [redacted]
✉ [redacted]

A Angaben zu Ihrer Person und Ihrer Lebenssituation

A1

Heutiges Datum, z.B. 01.08.23 ✎

Tag		Monat		Jahr	

Alter ✎ __________

Geschlecht ❑ weiblich ❑ männlich ❑ divers ❑ keine Angabe

Familienstand

verheiratet	in eingetragener Partnerschaft lebend	geschieden/ getrennt	verwitwet	ledig, war nie verheiratet
❑	❑	❑	❑	❑

Falls Sie ledig, geschieden/getrennt oder verwitwet sind: Haben Sie derzeit eine feste Partnerschaft? ❑ ja ❑ nein

Haben Sie Kinder? ❑ ja, ich habe ✎______ Kinder (Anzahl) ❑ nein, ich habe keine Kinder

Wie viele Personen leben insgesamt in Ihrem Haushalt, Sie selbst eingeschlossen?
Zählen Sie dabei bitte auch Kinder mit und tragen Sie die Anzahl ein.

insgesamt ✎__________ Personen, davon ➔ ✎_________ unter 18 Jahre alt

Wie würden Sie Ihren Wohnort am ehesten einordnen? **Ich lebe…**

in einer Großstadt	in einer mittleren Stadt	in einer Kleinstadt	auf dem Land
❑	❑	❑	❑

Welcher Glaubensrichtung/Konfession fühlen Sie sich zugehörig?

❑ christlich ❑ islamisch ❑ buddhistisch ❑ hinduistisch ❑ jüdisch

❑ keiner ❑ andere, und zwar: ✎______________________________

Manche Menschen sind nicht in Deutschland geboren, manche haben die Staatsangehörigkeit eines anderen Landes, wieder andere sind in Deutschland geboren, nachdem ihre Eltern nach Deutschland gezogen sind. Wir möchten gerne erfahren, ob etwas davon bei Ihnen zutrifft.

	**ja**	**nein**	**keine Angabe**
Ich besitze die deutsche Staatsangehörigkeit.	❑	❑	❑
Ich wurde außerhalb Deutschlands geboren.	❑	❑	❑
Meine Mutter und/oder mein Vater sind außerhalb Deutschlands geboren und erst nach 1949 nach Deutschland zugewandert.	❑	❑	❑

A2

Welches ist Ihr höchster allgemeinbildender Schulabschluss?	Welches ist Ihr höchster berufsqualifizierender Abschluss?
❑ noch kein Schulabschluss	❑ noch in beruflicher Ausbildung
❑ Schule beendet ohne Abschluss	❑ kein Berufsabschluss
❑ Haupt-/Volksschulabschluss	❑ abgeschlossene Lehre (beruflich-betriebliche Ausbildung)
❑ Realschulabschluss/Mittlere Reife/ Fachschulreife	❑ Handelsschule/Berufsfachschule (beruflich-schulische Ausbildung)
❑ Abschluss der Polytechnischen Oberschule 10. Klasse (vor 1965: 8. Klasse)	❑ Abschluss an Fachschule, Meister-, Technikerschule, Berufs- oder Fachakademie
❑ Fachhochschulreife	❑ Fachhochschulabschluss/Ingenieurschule
❑ allgemeine/fachgebundene Hochschulreife/ Abitur	❑ Abschluss an Universität/Hochschule
❑ anderer: ✎____________________	❑ anderer: ✎________________________

A3 Welche berufliche Position nehmen Sie ein?
(Falls Sie arbeitslos, berentet oder aus anderen Gründen erwerbsunfähig sind, geben Sie bitte Ihre **letzte Position** an.)

**Arbeiter:in**	**Angestellte:r** mit	**Beamt:in**
❑ Ungelernte:r	❑ einfacher Tätigkeit (z.B. Verkäufer:in, Kontorist:in)	❑ im einfachen Dienst
❑ (An-)Gelernte:r	❑ qualifizierter Tätigkeit (z.B. Sachbearbeiter:in) oder angestellte:r Industrie-/Werkmeister:in	❑ im mittleren Dienst
❑ Facharbeiter:in	❑ hochqualifizierter Tätigkeit oder Leitungsfunktion (z.B. Abteilungsleiter:in)	❑ im gehobenen Dienst
❑ Vorarbeiter:in, Meister:in, Polier:in	❑ umfassender Führungstätigkeit und Entscheidungsbefugnissen (z.B. Geschäftsführer:in, Direktor:in)	❑ im höheren Dienst

Selbständige:r
- ❑ selbstständige:r Landwirt:in bzw. Genossenschaftsbauer:in
- ❑ freiberuflich selbstständige:r Akademiker:in
- ❑ Selbstständige:r im Handel, Gewerbe, Handwerk, Industrie, Dienstleistung
- ❑ Mithelfende:r im Familienbetrieb

} Anzahl Mitarbeiter:innen/ Partner:innen: ✎ ________

❑ **sonstige, hier nicht aufgeführte Position:** ✎ ______________________________

❑ **noch <u>nie</u> berufstätig gewesen** (z.B. Hausfrau/-mann; Schüler:in, Student:in oder Auszubildende:r)

A4 Wie ist Ihre aktuelle Arbeitssituation?
(Mehrere Kreuze sind möglich, z.B. berentet und geringfügig beschäftigt)

- ❑ berufstätig, in Vollzeit
- ❑ berufstätig, in Teilzeit
- ❑ berufstätig, geringfügig beschäftigt (520€-Job)
- ❑ arbeitslos
- ❑ berentet/pensioniert
- ❑ Hausfrau/-mann
- ❑ Schüler:in, Auszubildende:r, Student:in
- ❑ andere: ______________________

A5 An wie vielen Arbeitstagen waren Sie in den letzten 12 Monaten durch einen Arzt krankgeschrieben (arbeitsunfähig)?
Bitte auch Tage berücksichtigen, an denen Sie ggf. stationär in einem Krankenhaus aufgenommen waren.

❑ ungefähr ✎ [| |] Arbeitstage ❑ gar nicht ❑ trifft nicht zu *[nicht berufstätig]*

Falls Sie aktuell berufstätig sind: Sind Sie derzeit krankgeschrieben (arbeitsunfähig)?

❑ ja, seit ✎ Tag [|] Monat [|] Jahr [|] ❑ nein ❑ trifft nicht zu *[nicht berufstätig]*

A6 Wie hoch ist das monatliche Nettoeinkommen Ihres Haushalts insgesamt?
Nettoeinkommen: Die Summe aus Lohn/Gehalt/Einkommen usw. nach Abzug von Steuern und Sozialabgaben.

- ❑ unter 1250 €
- ❑ 1250 € bis unter 1750 €
- ❑ 1750 € bis unter 2250 €
- ❑ 2250 € bis unter 3000 €
- ❑ 3000 € bis unter 4000 €
- ❑ 4000 € bis unter 5000 €
- ❑ 5000 € oder mehr

A7 Sind Sie derzeit Hauptverdiener:in Ihres Haushalts?

❑ ja ❑ nein

B Angaben über den/die Patient:in aus Ihrer Angehörigensicht

B1 In welcher Beziehung stehen Sie zum/zur Patient:in? **Der/die Patient:in ist mein:e…**

- ❑ Ehepartner:in (verheiratet)
- ❑ Lebenspartner:in (nicht verheiratet)
- ❑ ehemalige:r Partner:in (geschieden oder getrennt)
- ❑ Elternteil
- ❑ Schwester/Bruder
- ❑ Kind
- ❑ Freund:in
- ❑ anderes: ✎___________________

Wie lange besteht diese Beziehung schon? ✎________ Jahre

Leben Sie mit dem/der Patient:in in einem Haushalt zusammen?

- ❑ ja, wir leben in einem Haushalt
- ❑ nein, wir leben in getrennten Haushalten
- ❑ nein, der/die Patient:in lebt in einer Einrichtung (z.B. Pflegeheim)

Alter Patient:in (in Jahren): ❑ bis 30 ❑ 31-40 ❑ 41-50 ❑ 51-60 ❑ 61-70 ❑ 71-80 ❑ > 80

Geschlecht Patient:in: ❑ weiblich ❑ männlich ❑ divers ❑ keine Angabe

B2 Wie lange ist es her, dass die Erkrankung des/der Patient:in zum ersten Mal festgestellt wurde (Erstdiagnose)?

- ❑ unter 3 Monate
- ❑ 3 bis unter 6 Monate
- ❑ 6 bis unter 12 Monate
- ❑ 1 bis unter 2 Jahre
- ❑ 2 bis unter 5 Jahre
- ❑ 5 bis unter 10 Jahre
- ❑ 10 Jahre oder mehr

B3 Welche Absprachen bestehen mit dem/der Patient:in? Der/die Patient:in hat:

- ❑ Patient:innenverfügung
- ❑ Vorsorgevollmacht ➔ **wenn ja**: wer ist bevollmächtigt? ❑ ich selbst ❑ eine andere Person
- ❑ gesetzliche Betreuung ➔ **wenn ja:** wer ist Betreuer:in? ❑ ich selbst ❑ eine andere Person

B4 Bitte kreisen Sie bei den folgenden Fragen die Zahl zwischen 1 und 7 ein, die am besten zutrifft:

Wie würden Sie insgesamt den Gesundheitszustand des/der Patient:in während der letzten Woche einschätzen?

1 2 3 4 5 6 7

sehr schlecht ... ausgezeichnet

Wie würden Sie insgesamt die Lebensqualität des/der Patient:in während der letzten Woche einschätzen?

1 2 3 4 5 6 7

sehr schlecht ... ausgezeichnet

C Fragen zur Versorgung und Unterstützung des/der Patient:in

C1 Im Erkrankungsverlauf: Wurde der/die Patient:in vor dieser aktuellen Aufnahme irgendwann einmal in einer Form der spezialisierten Palliativversorgung versorgt?

Spezialisierte Ambulante Palliativversorgung (SAPV): leistet spezialisierte Palliativversorgung für Patient:innen, die einer Versorgung zu Hause (oder in Pflegeeinrichtungen) bedürfen und unterstützt deren Angehörige.

Palliativstation: leistet spezialisierte Palliativversorgung für Patient:innen und deren Angehörige in spezialisierten Abteilungen innerhalb eines Krankenhauses.

Multiprofessioneller Palliativdienst: leistet spezialisierte palliative Unterstützung und Mitbehandlung für Patient:innen und deren Angehörige in anderen Bereichen des Krankenhauses außerhalb der Palliativstation.

- ❑ nein, noch nie
- ❑ ja, und zwar: Mehrere Kreuze sind möglich. ➔
  - ❑ SAPV zu Hause oder im Pflegeheim (Spezialisierte Ambulante Palliativversorgung)
  - ❑ Palliativstation im Krankenhaus
  - ❑ Multiprofessioneller Palliativdienst auf einer anderen Krankenhausstation

C2 Wo wurde der/die Patient:in vor dieser aktuellen Aufnahme zuletzt versorgt?

- ❑ zu Hause ➔
  - ❑ **ohne** ambulanten Pflegedienst
  - ❑ **mit** ambulantem Pflegedienst
  - ❑ **mit** SAPV
- ❑ Pflegeheim ➔
  - ❑ **ohne** SAPV
  - ❑ **mit** SAPV
- ❑ Palliativstation im Krankenhaus
- ❑ andere Station im Krankenhaus, nämlich: ✎ ______________________________ ➔
  - ❑ **ohne** Multiprofessionellen Palliativdienst
  - ❑ **mit** Multiprofessionellem Palliativdienst
- ❑ anderes, nämlich: ✎ __________________________________

C3 Welche Pflege oder praktische Unterstützung haben Sie für den/die Patient:in in den letzten zwei Wochen geleistet?
Mehrere Kreuze sind möglich.

- ❑ im Haushalt, beim Einkaufen und Besorgungen (z.B. Medikamente)
- ❑ bei Verwaltung und Finanzen
- ❑ bei Fahrdiensten, Terminvereinbarungen und als Terminbegleitung (z.B. Arztbesuch)
- ❑ bei Alltagshandlungen (anziehen, essen, trinken, Medikamente einnehmen)
- ❑ bei der Körperpflege
- ❑ bei Pflegemaßnahmen (Verbände, Lagerung)
- ❑ weiteres: ✎ ____
- ❑ in den letzten zwei Wochen habe ich keine Pflege oder praktische Unterstützung geleistet

Wie viel Zeit nahm diese Pflege oder praktische Unterstützung ungefähr in Anspruch?

In den letzten zwei Wochen habe ich mich an ✎ _____ Tag(en) um den/die Patient:in gekümmert.

Der Umfang betrug ca. ✎ _____ Stunden pro Tag.

Angehörige leisten wichtige emotionale Unterstützung, stehen beratend zur Seite und begleiten den/die Patient:in (z.B. Gespräche über Sorgen und Ängste).

Wie viel Zeit nahm diese emotionale Unterstützung, Beratung und Begleitung ungefähr in Anspruch?

Der Umfang betrug ca. ✎ _____ Stunden pro Tag an ✎ _____ Tag(en) in den letzten zwei Wochen.

**C4**	Alles in Allem:<br>Wie gut fühlen Sie sich derzeit in Ihrer Rolle als Angehörige:r eines nahestehenden, unheilbar erkrankten Menschen vorbereitet?

überhaupt nicht gut	nicht sehr gut	etwas	ziemlich gut	sehr gut
❑	❑	❑	❑	❑

Gibt es etwas, auf das Sie in Ihrer Rolle gerne besser vorbereitet wären?

❑ nein ❑ ja**, und zwar**: 🖉 ______________________________

____

D Fragen zu Ihrem Befinden und möglichen Belastungen

8-Item Screening Tool for Family Caregiver Burden in Palliative Care (CAREPAL-8): Ullrich A, Bergelt C, Marx G, Daubmann A, Benze G, Heine J, et al. The CAREPAL-8: a short screening tool for multidimensional family caregiver burden in palliative care. BMC Palliat Care. 2024;23(1):195. doi: 10.1186/s12904-024-01480-w

Distress Thermometer and adapted Problem List: National Comprehensive Cancer Network. Distress management. Clinical practice guidelines. J Natl Compr Canc Netw. 2003;1(3):344-74. doi: 10.6004/jnccn.2003.0031; German: Mehnert A, Müller D, Lehmann C, Koch U. Die deutsche Version des NCCN Distress-Thermometers. Z Psychiatr Psychol Psychother. 2006;54(3):213-23. doi: 10.1024/1661-4747.54.3.213

Generalized Anxiety Disorder 7-item Scale (GAD-7): Spitzer RL, Kroenke K, Williams JB, Löwe B. A brief measure for assessing generalized anxiety disorder: the GAD-7. Arch Intern Med. 2006;166(10):1092-7. doi: 10.1001/archinte.166.10.1092; German: Löwe B, Decker O, Müller S, Brähler E, Schellberg D, Herzog W, et al. Validation and standardization of the Generalized Anxiety Disorder Screener (GAD-7) in the general population. Med Care. 2008;46(3):266-74. doi: 10.1097/MLR.0b013e318160d093

Patient Health Questionnaire depression module (PHQ-9): Kroenke K, Spitzer RL, Williams JB. The PHQ-9: validity of a brief depression severity measure. J Gen Intern Med. 2001;16(9):606-13. doi: 10.1046/j.1525-1497.2001.016009606.x; German: Löwe B, Spitzer RL, Gräfe K, Kroenke K, Quenter A, Zipfel S, et al. Comparative validity of three screening questionnaires for DSM-IV depressive disorders and physicians' diagnoses. J Affect Disord. 2004;78(2):131-40. doi: 10.1016/s0165-0327(02)00237-9

Short-Form Health Survey (SF-8): Ware JE, Kosinski M, Dewey JE, Gandek B. How to score and interpret single-item health status measures: a manual for users of the SF-8™ Health Survey. Lincoln, RI: Quality Metric Incorporated; 2001; German: Beierlein V, Morfeld M, Bergelt C, Bullinger M, Brähler E. Messung der gesundheitsbezogenen Lebensqualität mit dem SF-8. Diagnostica. 2012;58(3):145-53

E Fragen zu Ihrem sozialen Umfeld

Brief Assessment of General Family Functioning Scale (BAFFS): Mansfield AK, Keitner GI, Sheeran T. The Brief Assessment of Family Functioning Scale (BAFFS): a three-item version of the General Functioning Scale of the Family Assessment Device. Psychother Res. 2019;29(6):824-31. doi: 10.1080/10503307.2017.1422213; German: Spitzer C, Lübke L, Göbel P, Müller S, Krogmann D, Brähler E, et al. Die Erfassung des allgemeinen familiären Funktionsniveaus: Psychometrische Evaluation der deutschen Version der Brief Assessment of Family Functioning Scale. Psychother Psychosom Med Psychol. 2022;72(7):292-8. doi: 10.1055/a-1692-8763

Oslo Social Support Scale (OSLO-3): Meltzer H. Development of a common instrument for mental health. In: Nosikov A & Gudex C, editors. EUROHIS: Developing common instruments for health surveys. Amsterdam: IOS Press; 2003:35-47; German: Kocalevent RD, Berg L, Beutel ME, Hinz A, Zenger M, Härter M, et al. Social support in the general population: standardization of the Oslo social support scale (OSSS-3). BMC Psychol. 2018;6(1):31. doi: 10.1186/s40359-018-0249-9

F Fragen zu Ihren Unterstützungsbedürfnissen

Family Inventory of Needs (FIN): Kristjanson LJ, Atwood J, Degner LF. Validity and reliability of the family inventory of needs (FIN): measuring the care needs of families of advanced cancer patients. J Nurs Meas. 1995;3(2):109-26; German: Schur S, Neubauer M, Amering M, Ebert-Vogel A, Masel EK, Sibitz I, et al. Validation of the Family Inventory of Needs (FIN) for family caregivers in palliative care. Palliat Support Care. 2015;13(3):485-91. doi: 10.1017/S1478951514000261

F2 Haben Sie diesen Fragebogen alleine oder mit einer weiteren Person zur Unterstützung ausgefüllt?

- ❑ alleine
- ❑ mit einer weiteren Person

Vielen Dank für die Beantwortung des Fragebogens

und die Unterstützung unserer Studie!
