# Supplementary material for: Psychosocial Burden and Supportive Care Needs of Informal Caregivers in Specialist Palliative Care: Protocol of a Multicenter Longitudinal Cohort Study to Identify Trajectories and Validate the Multidimensional Screening Tool CAREPAL-8
Source: JMIR Res Protoc. 2026 Jul 31;15:e78076. doi: 10.2196/78076 (PMC13427073; doi:10.2196/78076)
Supplement: Multimedia Appendix 5 [file resprot-v15-e78076-s005.pdf]

Self-developed items used in the baseline questionnaire.

	Questionnaire/scale	Description	Analyses
State of health of the patient and conditions of care	Self-developed items	Item 1: Amount of months since the initial diagnosis.<br><br>Item 2: “How would you rate the patient's overall state of health during the last week?” This item is answered on a 7-point Likert scale ranging from “very poor” to “excellent”.<br><br>Item 3: “How would you rate the patient's overall quality of life during the last week?” This item is answered on a 7-point Likert scale ranging from “very poor” to “excellent”.<br><br>Item 4: “During the course of the disease: Has the patient received any form of specialist palliative care prior to this current admission?” This item is answered with “yes” or “no”. In case of “yes” there is a multiple choice of different forms of specialist palliative care.<br><br>Item 5: “Where was the patient last treated before this current admission?” A multiple choice of different locations of care as well as free text options are given as an answer.	Single item analyses.
